# Processing and Characterization of Cellulose Nanocrystals/Polylactic Acid Nanocomposite Films

**DOI:** 10.3390/ma8125447

**Published:** 2015-12-01

**Authors:** Erin M. Sullivan, Robert J. Moon, Kyriaki Kalaitzidou

**Affiliations:** 1 School of Materials Science and Engineering, Georgia Institute of Technology, 771 Ferst Drive N.W., Atlanta, GA 30332, USA; erin.sullivan@gatech.edu (E.M.S.); robertmoon@fs.fed.us (R.J.M.); 2 The Forest Products Laboratory, U.S. Forest Service, 1 Gifford Pinchot Drive, Madison, WI 53726, USA; 3 George W. Woodruff School of Mechanical Engineering, Georgia Institute of Technology, 801 Ferst Drive N.W., Atlanta, GA 30332, USA

**Keywords:** cellulose nanocrystals, polylactic acid, nanocomposite film

## Abstract

The focus of this study is to examine the effect of cellulose nanocrystals (CNC) on the properties of polylactic acid (PLA) films. The films are fabricated via melt compounding and melt fiber spinning followed by compression molding. Film fracture morphology, thermal properties, crystallization behavior, thermo-mechanical behavior, and mechanical behavior were determined as a function of CNC content using scanning electron microscopy, differential scanning calorimetry, X-ray diffraction, dynamic mechanical analysis, and tensile testing. Film crystallinity increases with increasing CNC content indicating CNC act as nucleating agents, promoting crystallization. Furthermore, the addition of CNC increased the film storage modulus and slightly broadened the glass transition region.

## 1. Introduction

There has been much interest in recent years to develop more environmentally friendly alternatives to currently used plastics in the packaging industry. Polymers like polylactic acid (PLA) have been of particular interest due to its high strength and modulus [1]. PLA is a thermoplastic, aliphatic polyester that can be derived from renewable resources such as starch or sugar cane [2]. PLA is a sustainable alternative to petroleum-based thermoplastics, such as polyethylene terephalate (PET) and polystyrene (PS), as it is biodegradable and environmentally friendly [2,3]. However, the slow crystallization rate of PLA hinders its ability to be used in scalable production [4]. Often introducing nanofillers will enhance crystallinity and crystallization rate. Cellulose and cellulose nanomaterials (CN) have been shown to act as nucleating agents, promoting crystallization in thermoplastics [4,5]. 

CNs are cellulose based nanoparticles isolated from bulk cellulose sources, such as wood or plants, and due to their unique combination of high mechanical and barrier properties, low density, low toxicity, and potential to be sustainably produced at industrial scale quantities at a reasonable price [6], CNs have attracted attention as a polymer reinforcement and in composite development for potential applications such as packaging, biomedical implants, and textiles [7,8,9]. One type of CN that is considered in the current study is cellulose nanocrystals (CNC), which are rod-like particles (3–20 nm in width and 50–500 nm in length) of highly crystalline cellulose (65%–90%) [7,8]. 

The incorporation of CNC in PLA has previously been explored through melt compounding coupled with the use of solvents, compatibilizers and chemical modification of the CNC [4,10,11,12,13,14,15,16,17]. The use of silylated CNC (s-CNC) [4,10] and plasticizers, such as glycerol triacetate (GTA) [11] and polyethylene glycol (PEG) [12], have been shown to enhance dispersion and minimize agglomerations in PLA composites. However, plasticizers and compatibilizers will also have an influence on the composite properties, where an increase of elongation at break, but decreases in the elastic modulus and tensile strength of PLA have been reported [12,13]. Additionally, the use of toxic solvents and time consuming functionalization steps are not ideal for creating scalable products. An alternative approach to incorporating CNC into PLA is melt compounding combined with liquid feeding [11,14]. CNC tend to agglomerate when dry due to their high surface area to volume ratio [14]. Therefore, direct liquid feeding of an aqueous CNC suspension is used to help minimize CNC aggregation [14]. When using this type of approach, it is important to consider the miscibility of the aqueous medium and the polymer. For example, in previous research a CNC/polyvinyl alcohol (PVOH) suspension was fed into an extruder with PLA; however, the immiscibility of PLA and PVOH led to phase separation and CNC was preferentially located in the PVOH phase [15].

The unique processing aspect of the current study is that it focuses on fabricating CNC/PLA films via a two-step process: (1) melt compounding using direct liquid feeding, followed by melt fiber spinning and (2) compression molding. By fiber spinning prior to compression molding, the CNC agglomerate size may be partly controlled and minimized. Fabrication of melt spun CNC/PLA fibers has previously been investigated and the composite fibers displayed enhanced thermal stability due to the hindered polymer chain mobility upon addition of CNC [16]; however, these fibers were not compression molded to make films. This additional step may alter thermal-mechanical properties. The compression molded films produced in the current study were examined by fracture surface morphology, crystallization behavior, thermo-mechanical properties, and mechanical properties. 

## 2. Experimental Section 

### 2.1. Materials 

The polymer matrix material used was polylactic acid (PLA, *M*_n_ = 1.42 × 10^4^ g/mol, 96% L-lactide and 4% D-lactide) in semicrystalline pellet form. PLA pellets (PLA 3051D) were purchased from Nature Works LLC, Minnetonka, MN, USA. 

Unmodified cellulose nanocrystals (CNC) were purchased from the University of Maine Process Development Center and manufactured at U.S. Forest Service’s Cellulose Nanomaterials Pilot Plant at the Forest Products Laboratory, Madison, WI, USA. The as-received suspension was 11.9 wt % CNC/water and the CNC were derived from dissolving pulp via sulfuric acid hydrolysis. The average length and width of the CNC were 6.4 ± 0.6 nm and 138 ± 22 nm, respectively [18].

### 2.2. Characterization 

Fracture surface morphology was examined using scanning electron microscopy (SEM, Phenom-World, Eidenhoven, The Netherlands), Phenom World G2 Pro, at an accelerating voltage of 5 kV. To eliminate charging, the samples were first coated with gold using a Cressington Sputter Coater 108 (model 6002, Ted Pella, Inc., Redding, CA, USA) at ~0.08 mbar and ~30 mA using a 30 s deposition time. The thermal transitions and crystallization behavior were investigated using thermogravimetric analysis (TGA), TGA Q500 (Thermal Analysis Instruments, New Castle, DE, USA), and differential scanning calorimetry (DSC), DSC Q2000 (Thermal Analysis Instruments). For TGA, 7–15 mg samples were prepared in platinum pans and heated from 30 to 600 °C in nitrogen at a rate of 5 °C/min. For DSC, 5–7 mg samples were prepared in standard Tzero aluminum pans, equilibrated at 20 °C, and held isothermal for 5 min. The samples were heated to 175 °C at a rate of 5 °C/min and then cooled to 20 °C at a rate of 5 °C/min. The degree of crystallinity was calculated using the first DSC heating cycle to capture the thermal history, a result of processing conditions. The crystal structure of the films was determined using wide-angle X-ray diffraction (WAXD, PANalytical, Almelo, The Netherlands). WAXD measurements were performed using an X’Pert Pro Alpha 1 diffractometer (Cu Kα radiation, λ = 1.541 Å). The diffraction patterns were collected from a 2θ angle of 8° to 40° with a step size of ~0.02. (The diffractometers were operated at 45 kV and 40 mA). 

Thermo-mechanical properties were investigated using dynamic mechanical analysis (DMA), DMA Q800 (Thermal Analysis Instruments). Rectangular specimens with a width of ~3 mm and gauge length of ~10 mm were equilibrated at 35 °C and held isothermal for 5 min. The samples were then heated at 3 °C/min to 100 °C. The tests were conducted at a frequency of 1 Hz and a strain amplitude of 12 μm. The tensile properties were examined using rectangular shaped specimens with a gauge length of 40 mm and a width of 10 mm. The rate of grip separation was 12.5 mm/min in accordance with ASTM D882 [19]. An Instron 33R4466 and a 10 kN load cell were used. 

### 2.3. Fabrication of Films 

The CNC/PLA composite films were fabricated via a scalable, two-step method. First, the as-received CNC/water suspension was combined with PLA pellets at appropriate ratios via melt blending with direct liquid feeding using a DSM 15cc compounder (vertical, co-rotating twin-screw microextruder, (Xplore Instruments, Galeen, The Netherlands) at a screw speed of 150 rpm and an operating temperature of 175 °C for approximately 3 min to produce a CNC/PLA mixture having specific CNC wt % of either 0, 1, 2 or 3 wt %. The CNC/PLA mixture was then extruded out of a 0.8 mm orifice at a 15 RPM pull-out rate and melt spun fibers of ~60–70 μm diameter (draw ratio of ~12). The advantages of melt spinning fibers prior to compression molding was to help minimize the size of CNC agglomerates and increase the CNC alignment along the fiber long axis. The CNC/PLA composite fibers were then randomly aligned and compression molded into films of ~110 μm thick using a manual four-column 12 ton Carver hydraulic press (model 4122) with controlled cooling. The fibers were then softened on the heated platens for ~3 min at 175 °C before a load of ~1 MPa was applied for 3 min. To minimize plastic deformation and thermal residual stresses, the samples were allowed to cool via ambient air cooling to below the glass transition temperature (~50 °C) before being removed from the mold.

## 3. Results and Discussion

### 3.1. Film Fracture Surface Morphology 

**Figure 1:**
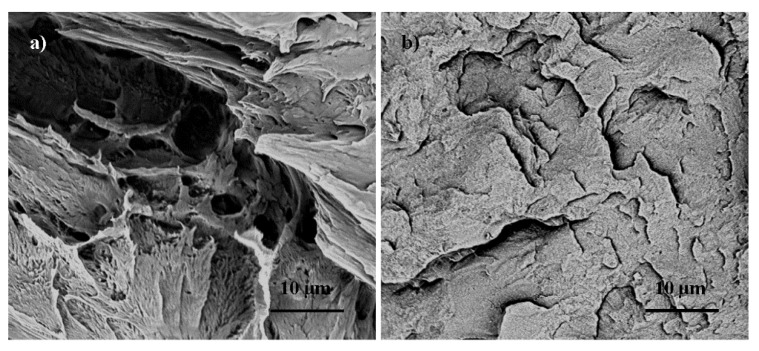
Representative scanning electron micrographs images of cryo-fracture surfaces for (**a**) 0 wt % cellulose nanocrystals/poly lactic acid composites (CNC/PLA) and (**b**) 3 wt % CNC/PLA composites.

Representative SEM images of the cryo-fracture surface of the 0 wt % and 3 wt % CNC/PLA films are shown in Figure 1. The flatter fracture surface of the composite film as compared to the neat PLA film is indicative of a more brittle fracture event, suggesting that the CNC additions are making the PLA more brittle. This embrittlement is similar to that reported by John *et al.* [16], in which melt spun fibers having 3 wt % CNC additions had a lower tensile strength and elongation at break as compared to the neat PLA fibers. There are many potential mechanisms for the property change, one that is considered here is that the CNC additions altered the crystallization behavior of the PLA [20]. CNC has been shown to act as a nucleating agent, promoting crystallization [4], in which changes in the extent of PLA crystallization would alter the fracture properties of the composite. To assess the role of CNC altering the PLA crystallization, crystallization behavior was examined. 

### 3.2. Crystallization Characterization of Composite Films 

**Figure 2:**
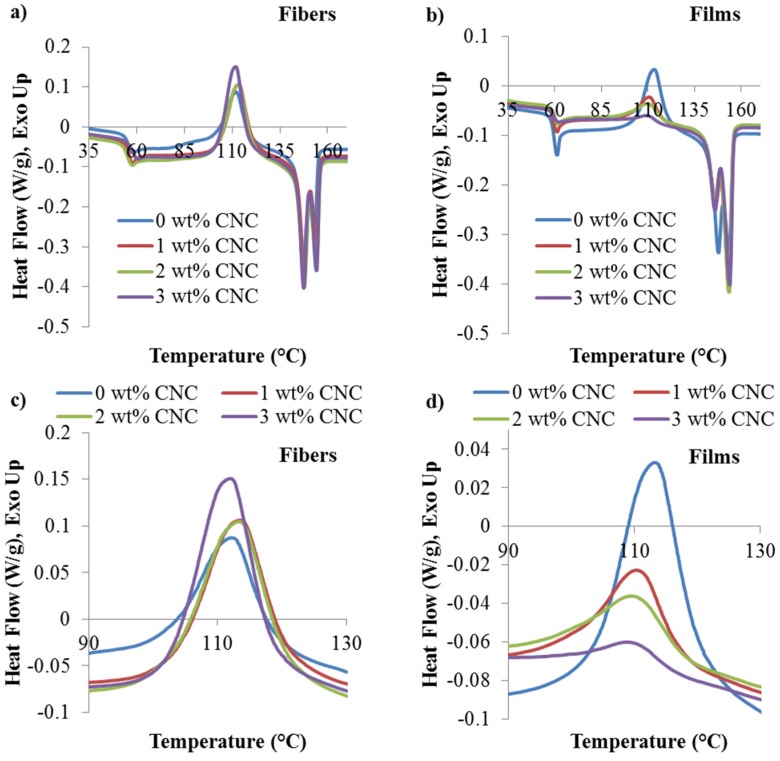
Non-isothermal initial heating thermograms of CNC/PLA (**a**) fibers; (**b**) films; (**c**) fiber cold crystallization peaks; and (**d**) film cold crystallization peaks.

Crystallization behavior of the CNC/PLA melt spun fibers and films were examined in order to investigate the effect of CNC on PLA crystallization characteristics. Initial heating thermograms, seen in Figure 2, were used to calculate the degree of crystallinity (χ) and examine the cold crystallization enthalpy (Δ*H_c_*) and cold crystallization temperature (*T_cc_*) as a function of CNC content for both the fibers and films. Equation (1) was used to calculate χ:

(1) $$\chi =\frac{\Delta H_{m}+\Delta H_{c}}{\Delta H_{m}^{{}^{\circ}}\left(1-wt\;\%/100\right)}\times 100$$

where χ is the degree of crystallinity, Δ*H_m_* is the melting enthalpy of the sample, Δ*H_c_* (<0) is the cold crystallization enthalpy of the sample, $\Delta H_{m}^{{}^{\circ}}$ is the theoretical melting enthalpy of 100% crystalline PLA, $\Delta H_{m}^{{}^{\circ}}$ = 93 J/g was used [21], and wt *%* is the CNC content in weight percent. The degree of crystallinity along with *|*Δ*H_c_|* and *T_cc_* is seen in Table 1. 

**Table 1:** Degree of crystallinity (χ), cold crystallization enthalpy (Δ*H_c_*), and cold crystallization temperature (*T_cc_*) for cellulose nanocrystals/poly lactic acid (CNC/PLA) fibers and films.

CNC content	χ (%)		|Δ*H_c_*| (J/g)		*T_cc_* (°C)	
	Fibers	Films	Fibers	Films	Fibers	Films
**0 wt % CNC**	5.7 ± 2.4	11.5 ± 0.8	22.7 ± 2.6	16.2 ± 2.1	113.2 ± 0.5	113.0 ± 0.5
**1 wt % CNC**	2.2 ± 2.1	24.1 ± 0.2	28.0 ± 2.9	7.3 ± 1.3	113.7 ± 0.2	110.2 ± 0.1
**2 wt % CNC**	1.6 ± 1.2	25.3 ± 0.2	28.2 ± 2.8	5.8 ± 0.7	114.5 ± 1.4	109.9 ± 0.2
**3 wt % CNC**	1.3 ± 1.3	29.7 ± 0.5	29.8 ± 0.7	1.9 ± 0.2	113.2 ± 1.3	109.3 ± 0.1

The fibers are highly amorphous with large |Δ*H_c_|* because the PLA chains do not have ample time to organize into crystalline lamella due to the rapid cooling of the fibers during spinning. However, the films’ degree of crystallinity is higher than that of the fibers and also increases with increasing CNC content due to the slow cooling rate during compression molding allowing time for crystallization coupled with CNC acting as a nucleating agent. For example, the 0 wt % CNC film has a relatively low crystallinity at approximately 11.5%, while the 3 wt % CNC film has a relatively high crystallinity at approximately 29.7%. Furthermore, both the |Δ*H_c_|* and *T_cc_* decrease with increased CNC content, seen in Figure 2c,d and displayed in Table 1. This increase in χ and decrease in both |Δ*H_c_|* and the cold crystallization peak as a function of CNC content indicates that the CNC are acting as a nucleating agent, promoting crystallization in the PLA films [4]. As seen in Figure 2a,b, the melting endotherms for both the fibers and films are comparable and do not change with increased CNC content. Both the fibers and films displayed double melting behavior, which is indicative of either polymorphism or melt recrystallization. Polymorphism refers to when there are multiple crystal phases present in a sample and melt recrystallization occurs when semi-melted crystals present recrystallize instead of melting further. To further investigate the crystal structure of the composite fibers and films, diffraction patterns were examined.

**Figure 3:**
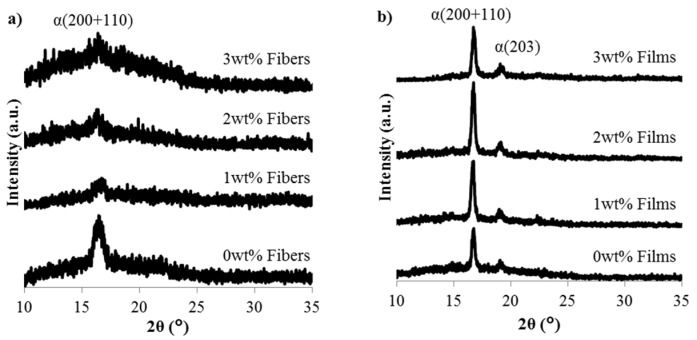
Diffraction patterns obtained for the CNC/PLA (**a**) fibers and (**b**) films as a function of CNC content.

PLA is comprised of three primary crystal phases: α, β, and γ [22]. The pseudo-orthorhombic, helical α phase crystal is the most stable and prevalent of the three primary crystal phases [22,23,24]. The diffraction patterns of the CNC composite fibers and films are seen in Figure 3. Both the fibers and films were comprised of primary *α* phase crystals and do not display evidence of a secondary crystal phase, therefore the double melting behavior seen in Figure 2 is due to melt recrystallization and not polymorphism. The diffraction patterns of the fibers, seen in Figure 3a, display a broad maximum at 2θ = 16.7° corresponding to the characteristic plane of α (200 + 110) [24] and this is in good agreement with the low crystallinity calculated using Equation (1) and displayed in Table 1. The CNC films display characteristic peaks of crystalline PLA at 2θ = 16.7° and 19.1° which correspond to the *α* phase crystalline peaks with characteristic planes of (200 + 110) [24] and (203) [25], respectively. 

**Figure 4:**
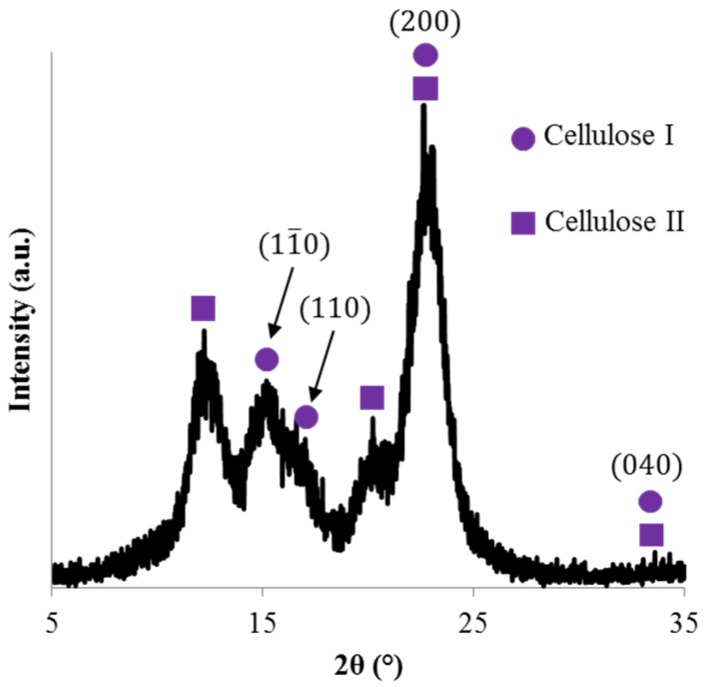
Diffraction pattern of a CNC mat with cellulose I and cellulose II primary diffraction peaks indicated.

The diffraction behavior of both the composite fibers and films is dominated by the PLA phase, which is the primary phase of the composite. In order to ascertain the crystal structure of the reinforcing phase, the as-received CNC/water suspension was dried in a vacuum oven and the diffraction pattern of the resultant CNC mat is shown in Figure 4. The CNC display primary peaks at 2θ = 15.1°, 17.5°, and 22.7° with a weak diffraction peak at 34.4° which correspond to the cellulose I crystal planes (1–10), (110), (200), and (040), respectively [10,26,27]. Diffraction peaks are also seen at 2θ = 12.5° and 20.1° which is consistent with the primary peaks associated with cellulose II which are at 2θ = 12.5°, 20.1°, 22.7°, and 34.4° [27]. The obtained crystal structure of the CNC mat is consistent with previously reported highly crystalline CNC of both cellulose I and II [10,26,27]. The existence of cellulose II in the CNC is typical of CNC from Forest Products Laboratory and is likely an artifact of using dissolving pulp as the source materials [28], but may also be attributed to the acid hydrolysis extraction method and exposure to alkali and acid treatments [27].

**Figure 5:**
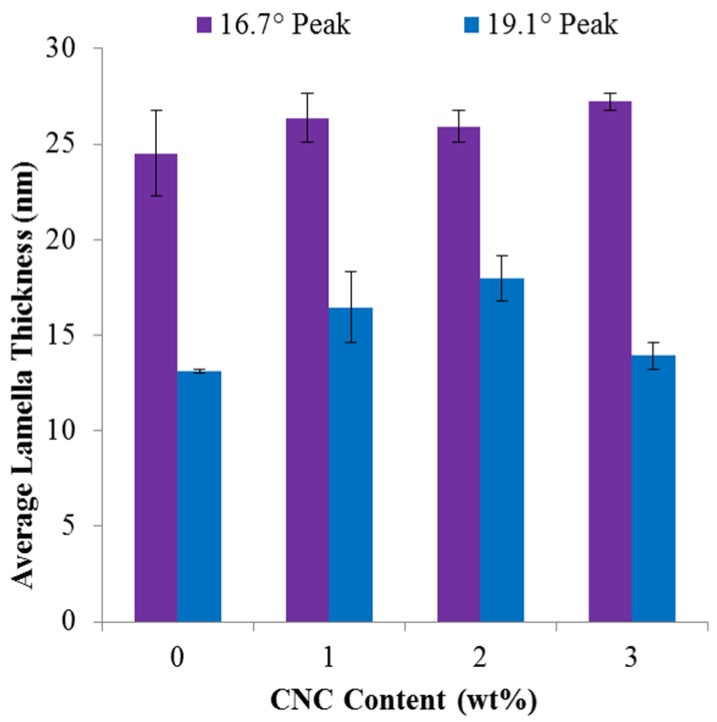
Average crystal lamella thickness of the two dominant diffraction peaks, 16.7° and 19.1°, for the CNC/PLA films as a function of CNC content.

To further investigate the crystal structure of the composite films as a function of CNC content, the average lamella thickness at the PLA primary diffraction peaks, 16.7° and 19.1°, was calculated using the Scherrer equation, Equation (2):

(2) $$L=\frac{K\lambda }{B\cos\left(\theta \right)}$$

where *L* is the apparent crystalline lamella thickness, *K* is a dimensionless shape factor (0.9 was used as an approximate for the spherulite structure [29]), λ is the radiation wavelength, *B* is the full width at half maximum value of the diffraction peak, and θ is the Bragg angle. As seen in Figure 5, the average lamella thickness does not significantly change for either primary diffraction peak as a function of CNC content. However, the degree of crystallinity significantly increases as a function of CNC content, as reported in Table 1, indicating that while the crystallinity of the films is increasing the average spherulite size is constant. Therefore, as the CNC content increases, there are more spherulites of comparable size present.

### 3.3. Thermo-Mechanical and Mechanical Response of Composite Films 

**Figure 6:**
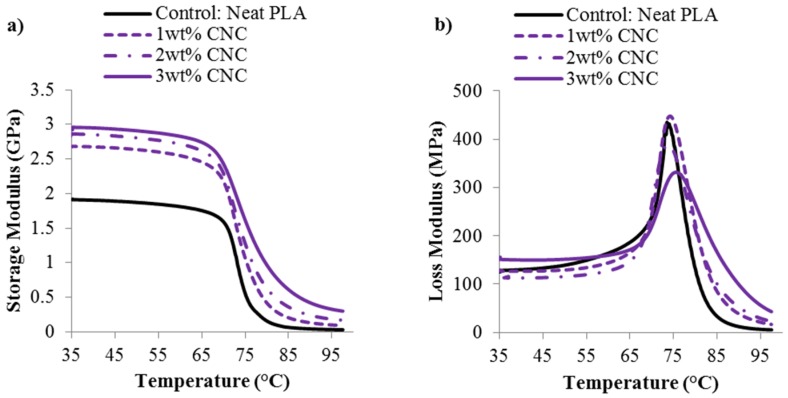
(**a**) Storage modulus and (**b**) loss modulus of the CNC/PLA films as a function of CNC content.

The thermo-mechanical behavior of the CNC/PLA films, both the storage modulus (*E′*) and loss modulus (*E′′*), was investigated as a function of CNC content. The results are seen in both Table 2 and Figure 6. The storage modulus increases significantly from 1.9 ± 0.3 GPa to 2.7 ± 0.0 GPa with the addition of only 1 wt % CNC. An increase in storage modulus, can be attributed to hindered polymer chain mobility due to: (1) an increase in polymer crystallinity [30]; (2) the addition of CNC constricting polymer chain movements [31,32]; or (3) a combination of (1) and (2). In this case, the significant increase in storage modulus can be attributed to a combinatory effect because the degree of crystallinity steadily increases with the addition of CNC, as shown in Table 1. Furthermore, as displayed in Table 2, there is a slight increase in glass transition temperature (*T_g_*) with the addition of 3 wt % CNC. The *T_g_* values reported in Table 2 are from DMA measurements, specifically the tanδ peak which is defined as *E′′/E′*. It was difficult to ascertain whether there was a trend in *T_g_* from the initial heating thermograms; however, this may be due to the sensitivity of the measurement technique, *i.e.*, DSC [33]. Additionally, the loss modulus peak shows some indication of broadening upon the addition of CNC, particularly at a higher CNC content of 3 wt %. This can be attributed to the increase in PLA matrix crystallinity coupled with the addition of CNC, which will further hinder polymer chain mobility resulting in a slight broadening the glass transition region [17].

**Table 2:** Thermo-mechanical behavior of CNC/PLA films as a function of CNC content

CNC content	*E′* at 35 °C (GPa)	*T_g_*, from tanδ (°C)
**0 wt % CNC**	1.9 ± 0.3	79.3 ± 0.8
**1 wt % CNC**	2.7 ± 0.0	79.5 ± 0.3
**2 wt % CNC**	2.7 ± 0.1	78.8 ± 0.0
**3 wt % CNC**	2.9 ± 0.1	82.2 ± 0.3

**Figure 7:**
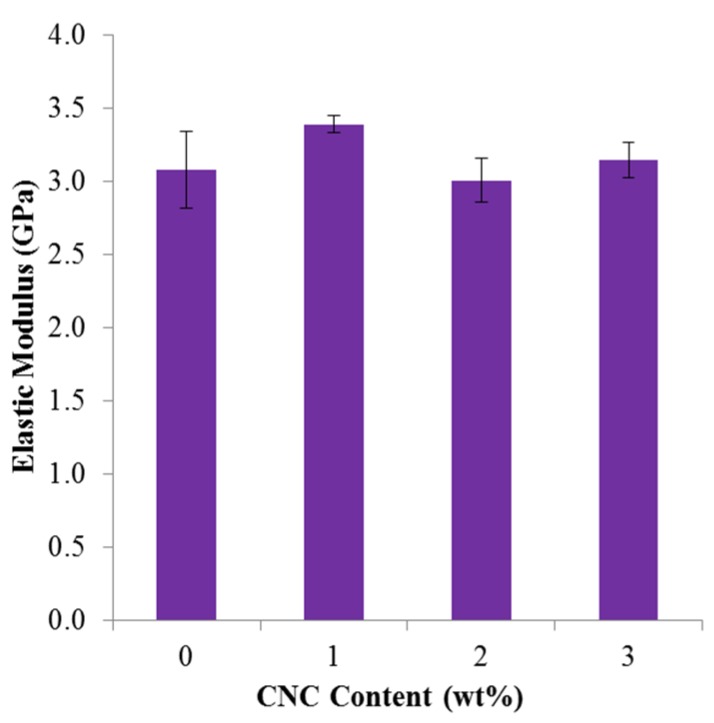
Elastic modulus of CNC/PLA films as a function of CNC content.

**Figure 8:**
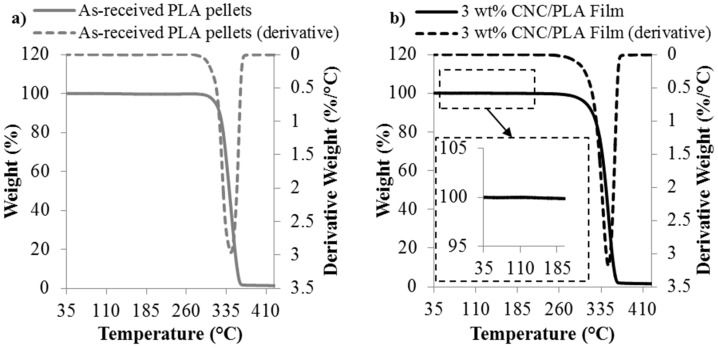
Representative results of the thermogravimetric analysis (TGA) of the (**a**) as-received PLA pellets and (**b**) 3 wt % CNC/PLA film.

The elastic modulus, *E*, of the composite films as a function of CNC content is shown in Figure 7. As seen in Figure 7, there is minimal change in elastic modulus as a function of CNC content for the CNC/PLA films. This is similar to that reported by John *et al.* [16], in which melt spun fibers having 3 wt % CNC additions had a modest 0.2 GPa increase in elastic modulus as compared to the neat PLA fibers. The previously reported elastic modulus of CNC (in the axial direction is ~60–105 GPa [34] and 20–50 GPa [35] in the transverse direction) is higher than that of PLA (~3 GPa), therefore, an increase in elastic modulus upon addition of CNC would be expected. However, while CNC has a higher intrinsic elastic modulus, there are several factors that may influence the modulus of the composites including: (1) the CNC/polymer interfacial interactions; (2) CNC distribution and dispersion; (3) CNC alignment; and even (4) moisture content in the composite [7]. Poor interfacial adhesion may also lead to poor nanofiller distribution and dispersion, which has previously been reported for CN/PLA composites fabricated via melt compounding [36]. Furthermore, because the CNC/PLA films were fabricated using direct liquid feeding of the as-received CNC/water suspension, TGA was performed on the films to investigate whether any moisture was present and representative results of the 3 wt % CNC/PLA films are seen in Figure 8. There is no significant moisture content present; therefore excess moisture is not a dominating factor to the plateau in elastic modulus with the addition of CNC content. Furthermore, due to the introduction of water during processing, the degradation of the 3 wt % CNC film was compared to the as-received PLA pellets. As seen in Figure 8a,b, the thermal stability of the 3 wt % CNC film is comparable to the as-received PLA pellets and does not appear to be compromised.

## 4. Conclusions 

Cryo-fracture surface SEM images indicated a more brittle fracture surface morphology upon addition of CNC when compared to neat PLA. This can be attributed to the increase in polymer matrix crystallinity with the addition of CNC, indicating CNC act as nucleating agents, promoting crystallization. While the CNC did not significantly alter the PLA crystal structure or spherulite lamella thickness, the CNC used was highly crystalline and composed of both cellulose I and II types due to the acid hydrolysis extraction method. The storage modulus of the films did increase with the addition of CNC due to the increased polymer matrix crystallinity coupled with the hindered polymer chain mobility caused by the addition of nanofiller. There was not a significant change in elastic modulus with the addition of CNC which can be attributed to the: (1) CNC/polymer interfacial interactions; (2) CNC distribution and dispersion; and/or (3) CNC alignment.

The incorporation of CNC in PLA via melt compounding/compression molding to fabricate 100% biodegradable and biorenewable films using a scalable production method was explored. While the use of unmodified CNC did show promise to increase the crystallinity of PLA, the elastic modulus was not significantly altered. 
